# Self-Supervised Point Set Local Descriptors for Point Cloud Registration

**DOI:** 10.3390/s21020486

**Published:** 2021-01-12

**Authors:** Yijun Yuan, Dorit Borrmann, Jiawei Hou, Yuexin Ma, Andreas Nüchter, Sören Schwertfeger

**Affiliations:** 1 School of Information Science & Technology, ShanghaiTech University, Shanghai 201210, China; houjw@shanghaitech.edu.cn (J.H.); mayuexin@shanghaitech.edu.cn (Y.M.); soerensch@shanghaitech.edu.cn (S.S.); 2 Informatics VII—Robotics and Telematics, Julius-Maximilians-University Würzburg, 97070 Würzburg, Germany; borrmann@informatik.uni-wuerzburg.de

**Keywords:** point cloud registration, descriptors, self-supervised learning

## Abstract

Descriptors play an important role in point cloud registration. The current state-of-the-art resorts to the high regression capability of deep learning. However, recent deep learning-based descriptors require different levels of annotation and selection of patches, which make the model hard to migrate to new scenarios. In this work, we learn local registration descriptors for point clouds in a self-supervised manner. In each iteration of the training, the input of the network is merely one unlabeled point cloud. Thus, the whole training requires no manual annotation and manual selection of patches. In addition, we propose to involve keypoint sampling into the pipeline, which further improves the performance of our model. Our experiments demonstrate the capability of our self-supervised local descriptor to achieve even better performance than the supervised model, while being easier to train and requiring no data labeling.

## 1. Introduction

Point cloud registration (PCR) is an essential task in various applications, including 3D reconstruction and simultaneous localization and mapping (SLAM). Usually, the accuracy of the calculated transformation will dominate the performance of higher level tasks. Thus, researchers either make back-end optimization on the high level task, such as SLAM [1,2,3], or improve on the PCR side.

Various techniques have been invented for the point set registration problem. As discussed in [4], it is extremely hard to find the optimal transformation T and correspondence matrix P simultaneously. The problem is addressed in [4] by alternating the optimization of T and P. In recent decades, a multitude of algorithms have been proposed on 3D registration. They are divided into rigid and non-rigid algorithms [5] and work either iteratively to solve for the transformation matrix with repeatedly matched points [6,7,8,9,10] or treat the problem as an optimization program that omits the necessity of computing correspondences [11,12,13].

For large datasets, global optimization is inefficient. Iterative methods, such as the Iterative Closest Point algorithm (ICP) [7], are more practical. To find the correspondences, using distance among descriptors rather than Euclidean distance between points promises improvements in point cloud registration, especially when no good initial guess is available. Then, handcrafted descriptors [10,14,15,16,17,18,19,20] and learned descriptors [21,22,23,24,25,26,27] have been proposed during the last decades.

Although the learned point descriptors score better, their supervising usually requires extra labor to label the data. Those algorithms either get the correspondences from the matched point clouds [21,22,23,25], which is costly, or they are labeling the inter-point cloud relation [26,27], which is inefficient to train. Moreover, the existing models either train on patches, which is not globally learned for the scene [21,22,24], or learn the scene as the training loss works globally for the whole point cloud but comes with a triplet siamese loss that is not directly related to the true transformation [26].

In this paper, we propose to learn a point cloud local descriptor for registration without any annotation and selection of patches. The input of the network is a raw point cloud for each iteration of training. In addition, our loss function is directly related to the solved true transformation of registration. To realize the self-supervision, we propose the Full Connection Form Solution (CF) to solve the PCR problem non-iteratively in one-step without correspondences. Then, it serves as a layer of a neural network in the end of the descriptor, the gradients are propagated back to the descriptor learner. Moreover, in our model, we use a keypoint detector to sample points in the layer of sampling and grouping [28] to avoid learning on non-interested points, which further improves the performance.

To summarize, the major contributions of this paper are:

- We propose a self-supervised method to learn point cloud descriptors requiring no manual annotation and selection during training.
- We propose a keypoint sampling manner during training, which can focus on interesting points and further boost the performance.
- Experiments show that our self-supervised learned local descriptor has better performance than the supervised 3DFeatNet.

Experiments on various datasets, i.e., on the Oxford [29] and KITTI [30] datasets, demonstrate the performance of our descriptor.

## 2. Related Work

This section first reviews the technical advances in point cloud registration which are related to our registration layer. Then, it describes handcrafted registration descriptors and learned models.

### 2.1. Registration Model

The Iterative Closest Point (ICP) algorithm is the most famous registration method. It has been widely applied to various representations of 3D shapes [7] and is able to align a set of range images into a 3D model [8]. The generalized-ICP [9] even puts point-to-point ICP and point-to-plane ICP into one probabilistic framework. ICP consists of two steps, correspondence search and solving the transformation.

However, in ICP and related methods, the correspondences have to be recomputed each iteration. To avoid this, the kernel correlation (KC) method [11] uses an objective function that fully connects the point clouds. In each term of the summation, a robust function, the Gaussian distance, has been utilized. Similar to Maximum Mean Discrepancy (MMD), KC evaluates the distance between two distributions. Thus, it shows better sensitivity to noise and is more robust than ICP-like methods. Some recent publications do not rely on correspondences. Myronenko and Song [12] represented point clouds with Gaussian mixture models and solve the transformation by aligning the model centroids. Zheng et al. [13] built a continuous distance field for a fixed model and aligned the other point set model to minimize the energy iteratively. Yang et al. [31] reformulated the registration as a truncated least squares estimation (TEASER++), which is thus robust to many wrong correspondences. Resorting to frequency domain, Huang et al. [32] decomposed the registration problem of seven DoFs into multiple subproblems, which they solved with a closed-form solution.

Those methods either require correspondences, needed in frequency domain, or are solved iteratively, which cannot be applied as a differentiable layer in deep neural networks to solve the transformation without pre-knowing the match. Thus, we propose a registration layer to fill in this requirement.

### 2.2. Descriptors

Point Feature Histograms (PFH) are known as the most typical local 3D descriptors. They encode the geometrical properties of the neighborhood with a multi-dimensional histogram [14]. For real time application, Fast Point Feature Histograms (FPFH) break the full interconnection of neighbors in PFH. Thus, they achieve a linear time complexity and gradually have become the most commonly used handcrafted 3D descriptor [10]. Apart from the descriptors from point set geometry, spin images (SI) [15] and unique shape context (USC) [16] split the spatial space into bins and count the number of points in each as a histogram descriptor. In addition, the authors of [17,18] transformed local scans into range images to extract features. Flint et al. [19] proposed to extend the 2D-SIFT onto 3D images. Wu et al. [20] introduced a SIFT-like descriptor on projected 3D patches.

However, the correspondence from features requires a good distinctiveness of the descriptor, but the performance of descriptors usually varies on different point sets. Therefore, data-driven descriptors come into the view. 3DMatch proposes to learn a volumetric patch descriptor from correspondence labels [21]. Based on PointNet [33], PPFNet introduces a local descriptor that is highly aware of global context [23]. It learns from the truth correspondence matrix. With a voxelized smoothed density value representation, 3DSmoothNet also trains the network with a triplet of anchor and positive and negative samples [22]. Without using correspondence labels, PPF-FoldNet uses an encoder–decoder network to reconstruct the local patch fed in [24]. D3Feat proposes a joint learning of keypoint detector and descriptor [25]. The D3Feat provides descriptors and keypoint scores globally for all points, which introduces extra cost during inference. Thus, this method is also unable to provide descriptors solely for interested local patch.

As the training loss merely works on pairs of patches, the point-wise supervised models are not learning globally for the entire scene within the dataset. We classify the feature learning models into two groups, point-wise and point cloud-wise supervised models, on whether they learn directly from the relation between point clouds. For point cloud-wise supervised models, the training loss works globally for an entire point cloud, which is more related to the registration application. This intuition directs us to learn our model with only raw point clouds.

Weakly supervised on the positive/negative relation between point cloud frames, 3DFeatNet learns descriptors without explicitly specifying the correspondences [26]. As a by-product of its attention-aware loss function, keypoints are extracted by applying non-maximum suppression on the all points attentions. To tackle the speed issue, RSKDD proposes to use random sampling to replace the Farthest Point Sampling (FPS) of 3DFeatNet [27]. In addition, it embeds chamfer loss and point-to-point loss from the keypoint detection model USIP [34] to co-learn the keypoints and detectors. Since its learned descriptor is not for the cluster center but for shifted point instead, the detector and descriptor modules are not able to be decoupled. Therefore, 3DFeatNet provides a good basis to feed in whole point cloud as we demand. In addition, our model does not require any annotation and the loss function is directly on the solved transformation.

## 3. Method

In this section, the registration layer and keypoint sampling are introduced. Then, we demonstrate the whole training pipeline to learn the descriptor model.

### 3.1. The Registration Layer

We intend to use both full connection and the least squares form in this module. However, just replacing the kernel of KC with the quadratic distance will not work due to the distant pairs that would dominate the loss. As discussed in [11], the gradient of the quadratic function is very sensitive to outliers, so a more robust function, the Gaussian kernel, has been utilized. However, with Gaussian kernel, a solution in one step is impossible.

Thus, instead, our formula is a summation of weighted square distances for each fully connected point pair, which has a closed form solution for registration. Assume we have two point clouds P and Q with $p_{i}{\in P|}_{i\in \{1,\cdots N\}}$ and $q_{j}{\in Q|}_{j\in \{1,\cdots M\}}$. $p_{i},q_{j}\in R^{3}$. *N* and *M* are the number of points in P and Q, respectively. Then, the optimization task is

(1) $$\begin{array}{c} \min_{R,t}\sum_{i=1}^{N}\sum_{j=1}^{M}w_{i,j}||Rp_{i}+t-q_{j}{\left|\right|}^{2} \end{array}$$

where R, t are the rotation matrix and translation vector to transform P into the coordinate system of Q. The weight $w_{i,j}$ in range (0,1] will be assigned for each term.

The other problem of Gaussian kernel distances in the KC method is that σ in the Gaussian kernel has to be properly set according to the scale of different data sources. We use the square distance, as it is invariant to scale [35].

For the weighted function (1), there is a full connection with quadratic distance between every point p∈P and q∈Q. Then, Equation (1) is reformulated with full connection as correspondences. The new point sets (X,Y) are of size N×M and each pair is a connection. Let $X=\{p_{1}^{\prime },\cdots p_{NM}^{\prime }\}$, $Y=\{q_{1}^{\prime },\cdots q_{NM}^{\prime }\}$; the problem is formulated as

(2) $$\begin{array}{c} \left(R,t\right)=\underset{R\in SO\left(d\right),t\in R^{d}}{\mathrm{argmin}}\sum_{i=1}^{NM}w_{i}\left|\right|\left(Rp_{i}^{\prime }+t\right)-q_{i}^{\prime }{\left|\right|}^{2} \end{array}$$

with known weights $w_{i}>0$.

The optimal solution is obtained with any algorithm that computes the transformation. We choose the SVD [36], as also detailed by Sorkine [37]. To make the paper self-contained, we briefly discuss this in Appendix A. Following Sorkine [37], we obtain a closed form solution for above formula by using weighted SVD. However, to have the desired suppression effect of pairs, weights cannot be arbitrarily chosen. To determine the weights, we use $f_{X}\left(x\right)$ to denote a function that extracts a feature descriptor of the point x from the point cloud X. Then, the similarity is obtained as

(3) $$\begin{array}{c} w_{i}=e^{-\frac{1}{\beta }\left|\right|f_{X}\left(p_{i}^{\prime }\right)-f_{Y}\left(q_{i}^{\prime }\right){\left|\right|}^{2}}. \end{array}$$

The lower is the similarity, the lower is the weight of the pairs. Thus, the effect of the term on the objective function will be less. In this way, a pair of points with low similarity contributes only a little, as they have a large feature descriptor distance. The constant β in Equation (3) scales the feature distance. It depends on the selected feature descriptor.

More details and testing about this CF registration is provided in the Appendix A and Appendix B.

### 3.2. Keypoint Sampling

To learn with a whole point cloud as input, subsampling is a standard operation for PointNet-like model. 3DFeatNet uses FPS to sample points that are evenly distributed on the scene. RSKDD-Net uses random sampling to speed up on a large-scale dataset.

However, both sampling methods may result in the selection of non-interesting points, e.g., points that are not distinctive and do not contribute to the registration success, which requires to devote an extra pattern of features to those ordinary points. In the matching step, only interesting points are involved. It means that they waste both training power and feature space for non-interesting points.

Thus, in this work, we propose to use keypoint detectors in the sampling and grouping layer. Since the descriptors are learned for a specific detector, during inference, with the same detector, our descriptor scores better compared to the version with non-interesting points included.

**Figure 1:**
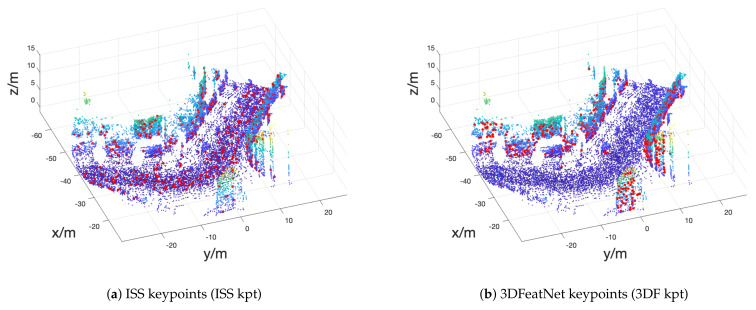
Keypoints demo. Two different keypoint detectors are applied to one selected Oxford frame, respectively: (left) ISS detector; and (right) 3DFeatNet Detector. Keypoints are plotted with red dots on point cloud.

We use one handcrafted ISS keypointer and learned 3DFeatNet keypoints (3DF kpt) because ISS are widely used handcrafted keypoints and 3DF kpt specially distributes points on the wall in Figure 1.

### 3.3. Network Architecture

**Figure 2:**
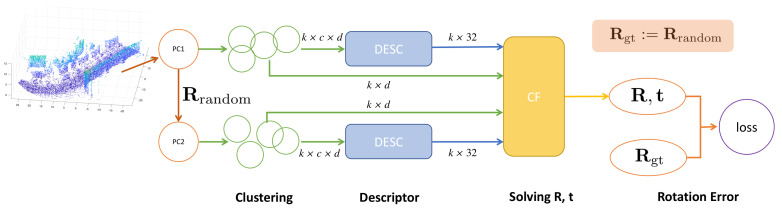
Pipeline of training: single input point cloud; branching with random rotation; clustering (sampling and grouping); descriptor; CF layer to solve for R,t and rotation error as the loss function.

We demonstrate the pipeline of the training process in Figure 2. The DESC module in between is the *f* we want to extract.

The whole training process consists of four parts. In the first stage, with a point cloud *PC1* as input, we apply a random transformation to generate *PC2*. For both *PC1* and *PC2*, we sample *k* points from a specific keypoint detector as centers. Then, neighbors are grouped around each center to obtain clusters. Then, those clustered are fed into the descriptor network *f*. Each cluster is processed separately and outputs a descriptor vector for the cluster center. Next, in the registration layer, CF (Section 3.1) solves Equation (2) for the transformation of sampled points with sampled centers and their descriptors from the two point clouds using the distance between the descriptors as weights according to Equation (3). The Rotation Matrix Distance Module computes the error between the solved R and $R_{\mathrm{gt}}$ considering the distance between the descriptors as weights, which is the loss function for our model.

Given the ground truth transformation $R_{\mathrm{gt}}$, $t_{\mathrm{gt}}$, the loss function is the deviation from the identity matrix [38] as follows

(4) $$loss=||I-RR_{\mathrm{gt}}^{T}{\left|\right|}_{F}.$$

When training the network, we only supervise the rotation because also involving the translation as a loss would further introduce additional hyperparameters to tune the balance between the effect from rotation and translation. Furthermore, the rotation is more important when performing the registration task.

With the above four parts of network components, the system merely requires to feed in one raw point cloud to learn for each iteration. Since the whole pipeline is differentiable, the parameters in the descriptor network are updated with gradient back-propagation. Given a random rotation, we minimize its distance to the solved rotation by optimizing *f*.

We call our model a self-supervised learning model because we generate labels ($R_{\mathrm{random}}$) from nothing and train the unlabeled data in a supervised way. The model is learned from a raw point cloud itself.

## 4. Experiment

### 4.1. Datasets

The Oxford RobotCar dataset [29] was used for network training and testing. Additionally, the KITTI dataset [30] was also used for testing the model.

#### 4.1.1. Oxford RobotCar Dataset

The Oxford dataset contains repeated traverses through the Oxford city center from May 2014 to December 2015 that were collected with the Oxford RobotCar platform. We used the pre-processed data from [26], which have 35 trajectories for training and another 5 trajectories for testing. The points were scanned from 2D LIDARs and are accumulated into 3D point clouds, using the GPS/INS poses. Those poses were refined with ICP. The training point clouds were then downsampled to about 50,000 ± 20,000 points and the test point clouds to exactly 16,384 points. In this way, 21,875 training and 828 testing point cloud sets were obtained.

#### 4.1.2. KITTI Dataset

Additionally, we tested our model on the 11 training sequences from the KITTI dataset [30] and processed them in the above-mentioned manner. The parts of the KITTI dataset used in the experiments include Velodyne laser point clouds, ground truth poses, and calibration files. The point clouds were also downsampled with a grid size of 0.2 m. We obtained 2369 point clouds in the end.

### 4.2. Setting

Our implementation makes use of the open source release (https://github.com/yewzijian/3DFeatNet) of 3DFeatNet [26]. In our pipeline, the descriptor directly uses the descriptor body of 3DFeatNet. Since this descriptor only considers a *z*-axis rotation, our provided $R_{\mathrm{random}}$ is generated by rotating around *z*-axis with $\phi ∼N(0,\sigma_{r}^{2})$. We used $\sigma_{r}=0.6$. In addition, we applied a 3D jitter with $\Delta p∼N(0,\sigma_{p}I)$ ($\sigma_{p}=0.01$) for each point in PC1 and PC2.

During the training of our model, we set batch size 6, Adam optimizer, and 32-dimensional descriptor. The training point clouds were randomly subsampled to 4096 points before feeding into the pipeline. We used the ISS and 3DFeatNet detectors (3DF kpt) to provide the keypoints as cluster centers to train. The setting of the 3DF kpt, e.g., $\beta_{\mathrm{attention}}$ and $r_{\mathrm{nms}}$, followed Yew and Lee [26]. We chose 256 keypoints from the point cloud to align the batch. We also used FPS to sample points as comparison. The FPS samples 512 points, which is the same as 3DFeatNet. In the cluster, each point is of dimension *d*, which can be 3 (xyz), 6 (xyzrgb), etc. We used d=3, thus we only used the xyz location of the point.

3DFeatNet states that it is hard to train. It takes 2 epochs to pretrain 3DFeatNet descriptor and the whole model can be trained in 70 epochs with $lr=10^{-5}$. In contrast, our network is easy to train: without any pre-training, our model is randomly initialized and saved at 10 or 20 epochs training with a learning rate $lr=10^{-3}$.

We used a PCL implementation of ISS to provide the ISS kpt and the released Tensorflow [39] checkpoint to achieve the network weight of 3DFeatNet to provide keypoints and run evaluation. We compared our method with handcrafted descriptors FPFH, SI, USC, and CGF and learned descriptors 3DMatch and 3DFeatNet.

### 4.3. Precision Test

**Figure 3:**
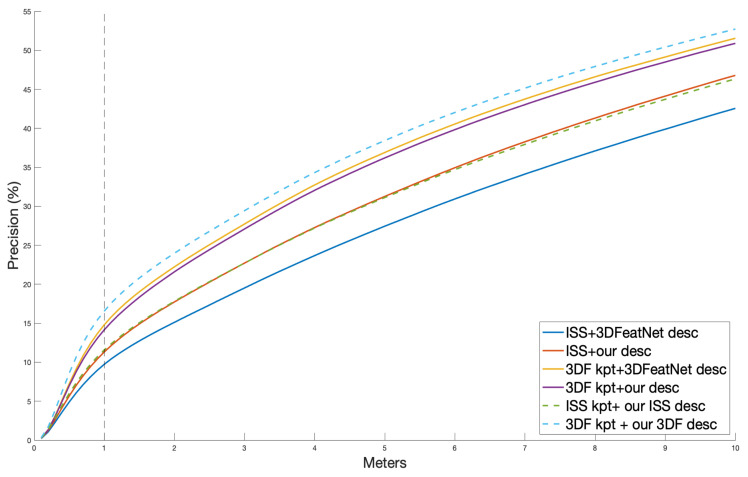
Precision plot for distance between nearest neighbor point and the ground truth location.

Using exhaustive search as in [26], this test searched for the nearest descriptor neighbor in the paired models for each keypoint. Then, the Euclidean distance between the neighbor location and ground truth location as computed. We show the plot in Figure 3. The *x*-axis is a threshold to consider a pair as correct and the *y*-axis is the correct proportion.

For both 3DfeatNet descriptor and our descriptor, the test with 3DF kpt works better than ISS kpt. Without using the keypoint sampling (with FPS instead), our proposed unsupervised model achieves a similar result to 3DFeatNet descriptor on 3DF kpt and a better result on ISS kpt. We used the x=1 m line as a cut. Both 3DFeatNet descriptor and our descriptor achieve around 15% precision, which is close to the best score in the record of Yew and Lee [26].

While using the keypoint sampling, we learned our ISS descriptor and our 3DF descriptor. ISS kpt + our ISS descriptor scores similar to our descriptor that used FPS. Both of our descriptors are better than 3DFeatNet descriptor on ISS keypoints. However, using ISS keypoint sampling during training does not improve our learned descriptor in the precision test. As shown in Figure 1, the ISS keypoints are evenly distributed in Oxford point cloud, which may introduce similar points as FPS. On the x=1 m line, with 3DF kpt, our 3DF descriptor learned the pattern and scores best. It is around 2% higher than the supervised descriptor.

Overall, our proposed unsupervised method scores better than the 3DFeatNet from which we borrow its descriptor part of model.

### 4.4. Geometric Registration

With ISS keypoints and 3DFeatNet keypoints, we evaluated the descriptors on the geometric registration. The registration uses nearest neighbor matches RANSAC to estimate the transformation. RANSAC iterations were limited to 10,000 and adjusted with 99% confidence. The Relative Rotation Error (RRE) and Related Translation Error (RTE), with respect to the ground truth, were computed to evaluate the accuracy of the registration. A success was decided when RTE < 2 m and RRE < 5 ${}^{\circ }$. The speed of converging was reflected by the average number of iterations. Since we used the same datasets (Oxford and KITTI) as 3DFeatNet experiment [26], we compared to the results from their table.

The evaluation on the Oxford data is demonstrated in Table 1. The first eight rows are taken from [26] and the last six rows are from our own experiments.

We observe that, firstly, except for PN++, the handcrafted descriptors cannot exceed the learned descriptors. Secondly, our unsupervised learned descriptor achieves the best result on RRE and the success rate with ISS and best result on RTE and average iteration with 3DF kpt. Thirdly, training merely on interested points, our keypoint sampling indeed improves the performance.

**Figure 4:**
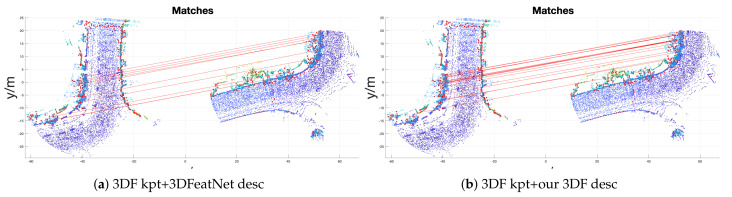
Oxford data geometric registration success sample. Keypoints are plotted with red dots on the point cloud. Red lines represent the matching between keypoints.

An example of a registration is shown in Figure 4. We observe that our 3DF descriptor has more inlier correspondences compared to 3DFeatNet descriptor by using 3DF keypoints, hich is revealed by the denser connection of the red lines.

Then, the model was transferred to another outdoor dataset, the KITTI dataset. The registration results are shown in Table 2. The first six rows of the results are taken from [26] and the last six rows are from our experiments.

**Figure 5:**
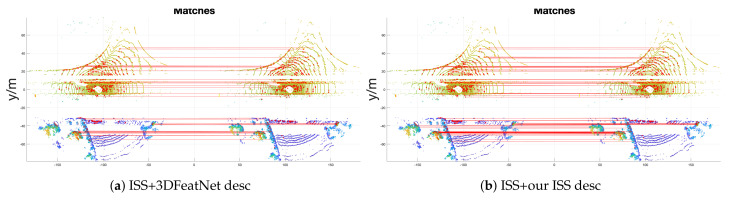
KITTI data geometric registration success sample. Keypoints are plotted with red dots on the point cloud. Red lines represent the matching between keypoints.

In the table, we observe that, firstly, our unsupervised model exceeds the supervised model. Secondly, ISS+our ISS descriptor achieves best accuracy. Its RRE even decreases about 0.041 compared to the FPS version (ISS + our descriptor). Thirdly, with 3DF kpt, our 3DF descriptor also achieves better results. A further example of a registration is shown in Figure 5. One can see that our ISS descriptor achieves much denser matching comparing to 3DFeatNet descriptor.

Overall, without using keypoint sampling, our unsupervised model achieves similar or even better performance than the supervised 3DFeatNet that uses the same descriptor body. In addition, with only interest points to train, our keypoint sampling indeed helps the model to learn more representative descriptors.

## 5. Conclusions

In this paper, we propose a novel self-supervised learning model to learn local descriptors for registration. We realize this goal by using a registration layer in the end. Thus, we use for supervision the randomly generated rotation of single point cloud input. In addition, we use keyopint sampling to make our model focus on interest points, in order to learn more expressive descriptors. In our pipeline, borrowing the same descriptor body as 3DFeatNet, our model is much easier to train, because this self-supervised method does not require any manual effort on annotation, and, without any pre-training, it converges with a higher learning rate, requiring far fewer iterations. Moreover, the experimental evaluation shows that our descriptor achieves much better performance on precision and geometric registration than the supervised 3DFeatNet descriptor.

As future work, we want to embedded our model into a SLAM framework to enable a no-annotation used data-driven descriptor for arbitrary scenes. 

## Figures and Tables

**Table 1:** Registration error on the Oxford dataset.

	RTE (m)	RRE (${}^{\circ }$)	Success Rate	Avg #Iter
ISS + FPFH	0.396	1.60	92.32%	7171
ISS + SI	0.415	1.61	87.45%	9888
ISS + USC	0.324	1.22	94.02%	7084
ISS + CGF	0.431	1.62	87.36%	9628
ISS + 3DMatch	0.494	1.78	69.06%	9131
ISS + PN++	0.511	1.88	48.86%	9904
ISS + 3DFeatNet desc	0.314	1.08	97.66%	7127
3DFeatNet kpt + 3DFeatNet desc	0.300	1.07	98.10%	2940
ISS + 3DFeatNet desc	0.314	1.08	97.66%	7126
ISS + our desc	0.311	1.01	98.10%	5648
ISS + our ISS desc	0.311	**1.00**	**98.23%**	5545
3DF kpt + 3DFeatNet desc	0.304	1.08	97.66%	3294
3DF kpt + our desc	0.310	1.08	97.05%	3650
3DF kpt + our 3DF desc	**0.298**	1.02	97.90%	**2703**

**Table 2:** Registration Error on the KITTI Dataset.

	RTE (*m*)	RRE (${}^{\circ }$)	Success Rate	Avg #Iter
ISS + FPFH	0.325	1.08	58.59%	7462
ISS + SI	0.358	1.17	55.92%	9219
ISS + USC	0.262	0.83	78.24%	7873
ISS + CGF	0.233	0.69	87.81%	7442
ISS + 3DMatch	0.283	0.79	89.12%	7292
3DF kpt + 3DFeatNet desc	0.258	0.57	**95.97**%	3798
ISS + 3DFeatNet desc	0.246	0.627	93.50%	8311
3DF kpt + 3DFeatNet desc	0.264	0.599	95.58%	4394
ISS + our desc	**0.215**	0.510	93.50%	5960
ISS + our ISS desc	**0.215**	**0.459**	93.85%	4356
3DF kpt + our desc	0.258	0.570	95.44%	3732
3DF kpt + our 3DF kpt	0.244	0.501	95.87%	**2631**

**Figure A1:**
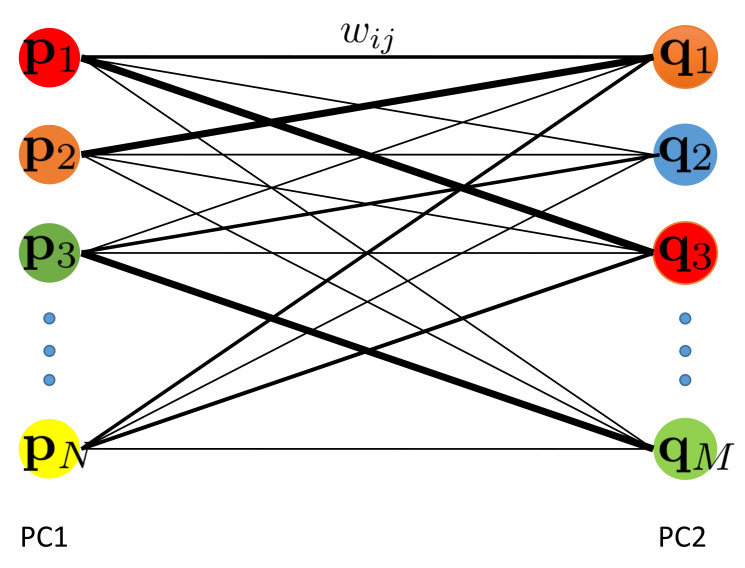
Full connection between two point sets. Each edge is a weighted Euclidean squared distance term in our object function, given a proper $w_{i,j}$ to scale the cost term of the pair (i,j). The thickness of the lines reflect the similarity (weight) of pairs.

**Figure A2:**
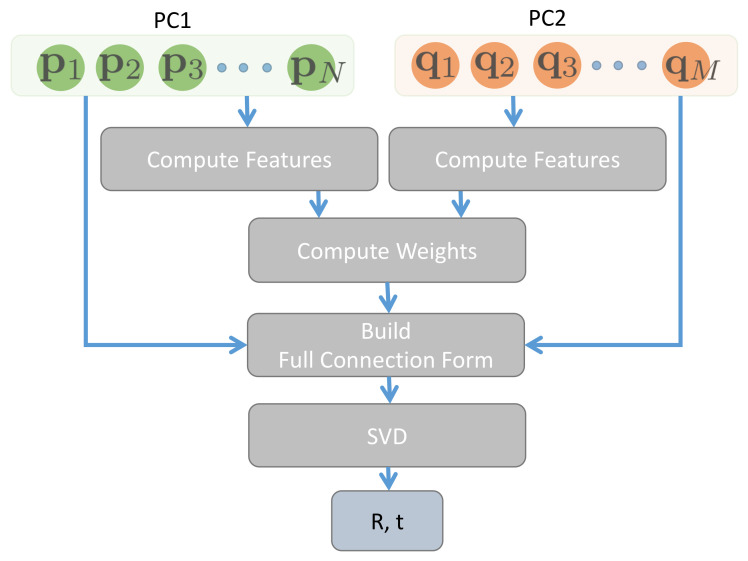
Schematic diagram of the CF registration model.

**Figure A3:**
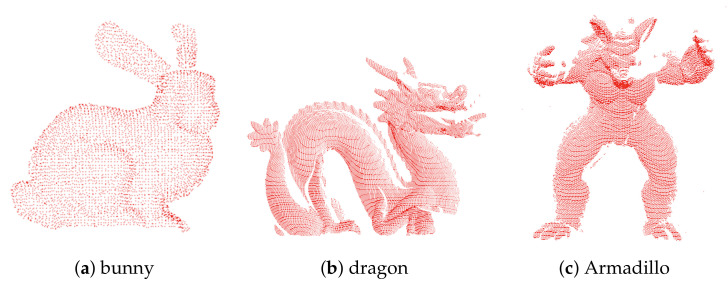
Three point cloud used for experiments.

**Figure A4:**
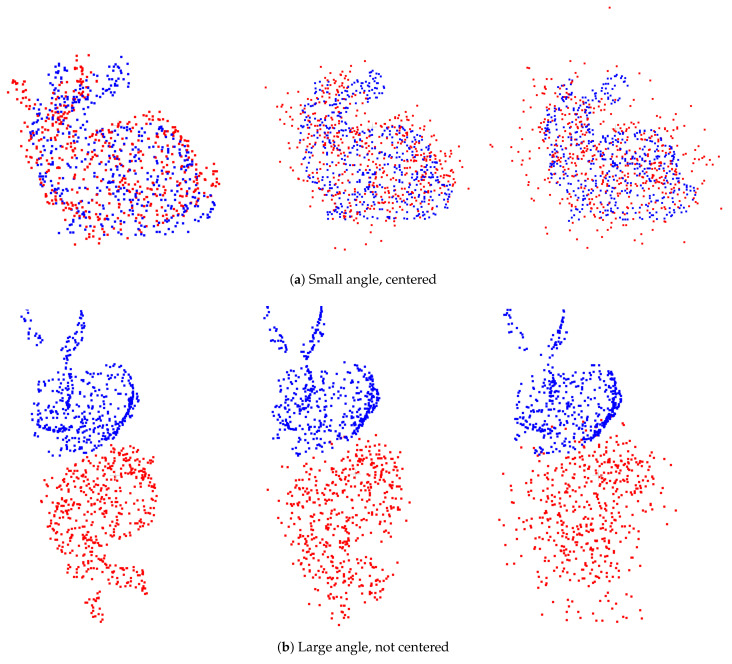
Noise data: (top) centered small angle; and (bottom) large angle. From left to right column is with noise standard derivation 0.002, 0.01, and 0.02.

**Figure A5:**
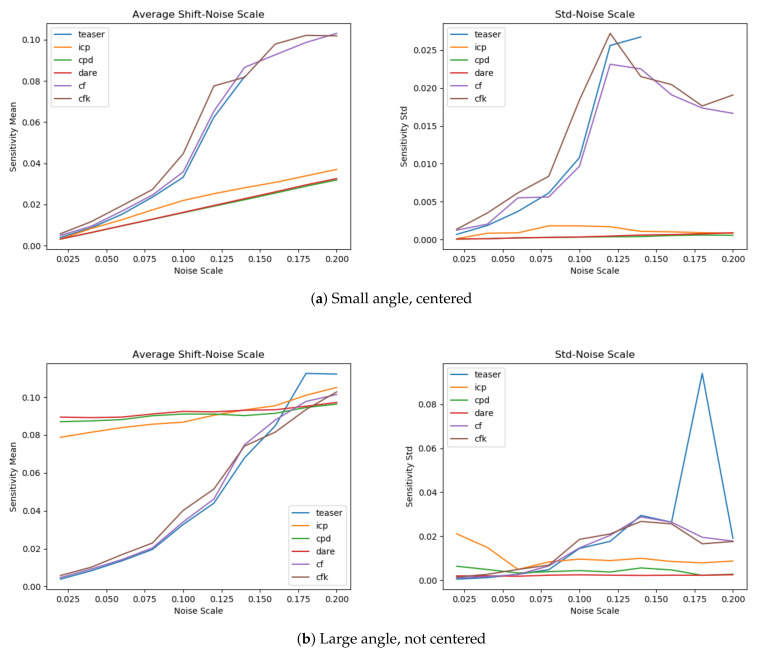
Sensitivity test. The left two plots show results with small rotation, centered. The right two plots show results with large rotation, not centered. The first and third diagrams show the mean shift to noise scale. The second and fourth diagrams show the standard deviation.

## Data Availability

Not applicable.

## Appendix A. CF Registration Model

The formula is a summation of weight square distances for each fully connected point pair. Figure A1 illustrates the full connection, where weights are set according to a similarity measure.

(A1) $$\begin{array}{c} \min_{R,t}\sum_{i=1}^{N}\sum_{j=1}^{M}w_{i,j}||Rp_{i}+t-q_{j}{\left|\right|}^{2} \end{array}$$

### Appendix A.1. Solving the Transformation

For the weighted function (A1), there is a full connection with quadratic distance between every point p∈P and q∈Q. Equation (A1) is reformulated with full connection as correspondences. The new point sets (X,Y) are of size N×M and each pair is a connection. Let $X=\{p_{1}^{\prime },\cdots p_{NM}^{\prime }\}$, $Y=\{q_{1}^{\prime },\cdots q_{NM}^{\prime }\}$; the problem is formulated as

(A2) $$\begin{array}{c} \left(R,t\right)=\underset{R\in SO\left(d\right),t\in R^{d}}{\mathrm{argmin}}\sum_{i=1}^{NM}w_{i}\left|\right|\left(Rp_{i}^{\prime }+t\right)-q_{i}^{\prime }{\left|\right|}^{2} \end{array}$$

with known weights $w_{i}>0$.

We cancel t by computing the weighted mean

(A3) $$\begin{array}{c} {\bar{p}}^{\prime }=\frac{\sum_{i=1}^{NM}w_{i}p_{i}^{\prime }}{\sum_{i=1}^{NM}w_{i}},{\bar{q}}^{\prime }=\frac{\sum_{i=1}^{NM}w_{i}q_{i}^{\prime }}{\sum_{i=1}^{NM}w_{i}} \end{array}$$

and centering the point clouds

(A4) $$\begin{array}{c} x_{i}:=p_{i}^{\prime }-{\bar{p}}^{\prime },y_{i}:=q_{i}^{\prime }-{\bar{q}}^{\prime }. \end{array}$$

We can then compute R

(A5) $$\begin{array}{c} R=\underset{R\in SO(d)}{\mathrm{argmin}}\sum_{i=1}^{NM}w_{i}||Rx_{i}-y_{i}{\left|\right|}^{2}. \end{array}$$

Let X denote the matrix where $x_{i}$ is the *i*th column. Similarly, we have Y. Thus, $X,Y\in R^{3\times NM}$. W is a diagonal matrix with $W_{i,i}=w_{i}$. The SVD solves it, where

(A6) $$\begin{array}{cc} U\Sigma V^{T} & =XWY^{T} \end{array}$$

and the optimal rotation is computed by

(A7) $$R=VU^{T}.$$

When the solution consists of a reflection, i.e., |V||U|<0, the last column of V will be multiplied by −1 before computing the rotation.

Finally, the translation is given as

(A8) $$\begin{array}{c} t={\bar{q}}^{{}^{\prime }}-R{\bar{p}}^{{}^{\prime }}. \end{array}$$

A schematic diagram to solve the registration is demonstrated in Figure A2.

### Appendix A.2. Weights as Similarity of Feature

To determine the weights, we use $f_{X}\left(x\right)$ to denote a function that extracts a feature descriptor of the point x from the point cloud X. Then, the similarity is obtained as

(A9) $$\begin{array}{c} w_{i}=e^{-\frac{1}{\beta }\left|\right|f_{X}\left(p_{i}^{\prime }\right)-f_{Y}\left(q_{i}^{\prime }\right){\left|\right|}^{2}}. \end{array}$$

The lower is the similarity, the lower is the weight of the pairs. Thus, the effect of the term on the objective function will be less. In this way, a pair of points with low similarity contributes only a little, as they have a large feature descriptor distance. The constant β in Equation (A9) scales the feature distance. It depends on the selected feature descriptor.

### Appendix A.3. Time Complexity

The runtime for the proposed method is dominated by two parts: computing the weights and solving the SVD. For convenience, we assume M=N. To compute the weight, point descriptors of each point cloud are computed, which takes O(NklogN), where *k* is the number of neighbors for each point. Then, setting up the $N^{2}$ weights takes $O(N^{2})$. In the SVD, we first compute the centroid and transform the point cloud to center, which takes $O(N^{2})$, because we have to consider NM terms. Since $W\in R^{NM\times NM}$ is a diagonal matrix, the multiplication for $XWY^{T}$ is equivalent to scaling each row *i* of $Y^{T}$ with $W_{i,i}$. Thus, to obtain $XWY^{T}$ takes $O(N^{2})$. As $XWY^{T}$ is a 3-by-3 matrix, solving the SVD costs only constant time.

Overall, the time complexity of the proposed method is $O(N^{2})$.

### Appendix A.4. A Variant: Applying on Point Set of Keypoints

For large point sets, the time complexity of $O(N^{2})$ becomes infeasible. One possible solution is to extract interest points and to apply the full connection cost to the two sets of keypoints.

For each point set with *N* points, using a handcraft keypoint detector, computing the normals takes O(NklogN) and keypoint detection takes O(N). Assume *n* points are extracted (n<<N), and then weight and SVD computation is done on *n* points. Overall, we yield $max(O\left(Nk\log N\right),O\left(n^{2}\right))$.

## Appendix B. Experiments and Results

We compare the proposed algorithm with ICP, a feature based state-of-the-art algorithm TEASER++ [31], Coherent Point Drift (CPD) [12] and Density Adaptive Point Set Registration (DARE) [40]. We call our method Full Connection Form Solution (CF) and CF-keypoint (CFK) (a variant with keypoints) for short.

In our experiments, the small 3D object datasets “bunny”, “dragon”, and “Armadillo” (bun000, dragonStandRight_0, and ArmadilloStand_180) from the Stanford website (http://graphics.stanford.edu/data/3Dscanrep/) were used. They are in bounding boxes with side lengths (0.156, 0.153, 0.118), (0.205, 0.146, 0.072), and (0.215, 0.275, 0.258) respectively. They are shown in Figure A3. With those, we evaluated our algorithms with respect to its sensitivity to noise, robustness to outliers, and accuracy of the registration.

### Appendix B.1. Settings

We first sampled the point clouds from the meshes using Meshlab [41]. For CPD, the open source C++ implementation from the original project [12] was used. We set its scale and reflection parameters to false. For DARE, we used the Python implementation of Järemo Lawin et al. [40]. Its color label and feature label were disabled. We also used TEASER++ from the implementation [31]. We implemented CF and CFK using the Point Cloud Library (PCL) [42], where we used its FPFH descriptor and the SIFT keypoint detector. The ICP experiments were also done with PCL. The normal and feature computation in CF and CFK were performed with the same settings, i.e., searching *k* neighbors. In our implementation, we fixed *k* to 150. In addition, the β used in Equation (3) was fixed to 100.

We set the ICP parameters with max correspondence distance 0.5, max iteration 1000, transformation epsilon 1 × 10${}^{-9}$, and Euclidean fitness epsilon 0.05.

For TEASER++, we used the same settings as for the feature descriptor FPFH. In the matcher of TEASER++, the options absolute_scale and crosscheck were selected. The solver used GNC_TLS with a 1.4 gnc factor, 0.005 rotation cost threshold, and 1000 max iterations.

In our experiments, the registration was done using two point clouds $PC_{a}$ and $PC_{b}$, which were generated with added noise or outliers from the original point cloud, as described in more detail below. We then translated and rotated $PC_{b}$ to get $PC_{b}^{{}^{\prime }}$. Thus, the $PC_{a}$ was our PC1 and $PC_{b}^{{}^{\prime }}$ was our PC2 and our task was to align PC1 to PC2 by solving for the transformation.

In the following experiments, $PC_{b}$ was transformed in two distinct ways to generate $PC_{b}^{{}^{\prime }}$. Firstly, we applied just a small, random rotation around the point clouds centroid. For the second type of data, we applied a large random rotation around the origin of the dataset, which is not the centroid.

The rotation vector is a concise axis–angle representation, for which both the rotation axis and angle are represented in the same three-vector. The rotation angle is the length of this vector.

The small rotation vectors have values drawn uniformly from [−π/8,π/8), while the large rotation vectors are uniformly drawn from [−π/2,π/2).

### Appendix B.2. Sensitivity to Noise

In this experiment, we evaluated the effects of different levels of noise on the registration. Each level was tested with 30 generated point clouds. Just for this experiment, we fixed the large and small rotation angles to two certain values, to be able to concentrate on the effects of the levels of noise and draw the diagrams of Figure A5. PC1 and PC2 used 500 points subsampled from the origin point cloud. Then, we rotated PC2 and added zero mean Gaussian noise to each point.

Following the definition of sensitivity [11], we logged the mean average shift to evaluate the performance, and the standard deviation was utilized as the metric. The noise scale was within the range (0,0.02]. Because the size of the bunny does not exceed 0.3, too large noise would result in dysfunctional feature descriptors. We present the noise data with different noise scales in Figure A4. The results are given in Figure A5. In the small angle case of Figure A5, the TEASER++ curve breaks due to a low number of correspondences and followed by failure.

For the centered small rotation, ICP, CPD, and DARE achieve better average shifts and less sensitivity to noise. For the feature based methods, our CF and CFK perform very similar to TEASER++.

However, for the large rotation data, ICP, CPD, and DARE fail to align the point clouds, while the feature-based methods CF, CFK, and TEASER++ are able to align with good performance.

### Appendix B.3 Robustness to Outliers

Similar to above, we also used 500 randomly selected points from the bunny object and performed small and large rotations. Additionally, 100 random points were uniformly drawn in a sphere and added to the rotated point set PC2 (with radius 0.2, around the center of sampled point clouds).

Because the first 500 points in each set are also from the same sampled index, we actually know the correspondence in the non-outlier parts. To quantify the robustness, we computed the average shift as in Appendix B.2.

For both large and small rotations, we tested 100 times to record the mean and standard deviation. The quantitive evaluation is given in Table A1. All experiments were made using randomly drawn rotation vectors.

CPD achieves extremely precise solutions for small rotations, while feature-based methods (TEASER++, CF, and CFK) are similar and better than ICP and DARE. DARE gives the largest error and standard deviation. For the large rotation case, the feature-based methods (TEASER++, CF, and CFK) perform best, and the errors of the remaining methods are several times worse and unstable, since they yield large standard deviations. The performance of our one-step methods is close to TEASER++, even though its truncated least squares is theoretically more insensitive to spurious data.

**Table A1:** Robustness test: smaller is better.

	Small Rotation, Centered	Large Rotation, Not Centered
ICP	0.0019±0.0062	0.070±0.023
CPD	2.4 × 10${}^{-9}$ ± 1.7 × 10${}^{-10}$	0.040±0.047
DARE	0.012±0.019	0.053±0.035
TEASER++	0.0055±0.0027	0.0057±0.0035
**CF**	0.0075±0.0028	0.0078±0.0032
CFK	0.0096±0.0043	0.0099±0.0052

### Appendix B.4. Accuracy

Using the same given transformation applied to the original point sets as in Appendix B.3, we achieve rotated models. Then we randomly sampled 500 points from both the reference model and the rotated models for testing. In the accuracy test, the three point sets in Figure A3 (bunny, dragon and Armadillo) were utilized. To evaluate the accuracy, deviations from the identity matrix [38] were computed:

$$ACC_{R_{\mathrm{gt}}}\left(R_{\mathrm{predicted}}\right)=||I-R_{\mathrm{predicted}}R_{\mathrm{gt}}^{T}{\left|\right|}_{F}$$

It is a distance measure using the Frobenius norm of a matrix, where $R_{\mathrm{gt}}$ is the given rotation and $R_{\mathrm{predicted}}$ is the predicted rotation.

Accuracy results are given in Table A2. For the centered small rotation case, we observe that CPD also achieves the best score while feature-based algorithms (TEASER++, CF, and CFK) are on the same level. For large rotations, CPD becomes unstable, which results in much larger average rotation distances and their standard deviations. The feature-based methods still show close results in different cases. Our one-step solution shows similar result to the truncated least squares method TEASER++.

**Table A2:** Accuracy test: smaller is better.

	Small Rotation, Centered			Large Rotation, Not Centered		
	Bunny	Dragon	Armadillo	Bunny	Dragon	Armadillo
ICP	0.045±0.018	0.045±0.19	0.036±0.022	0.96±1.14	1.09±1.13	1.17±1.15
CPD	0.016±0.0089	0.014±0.0083	0.012±0.0074	1.15±1.30	1.11±1.19	1.13±1.15
DARE	0.020±0.0093	0.016±0.0090	0.016±0.0083	1.30±1.26	1.34±1.22	1.48±1.16
TEASER++	0.14±0.076	0.15±0.084	0.16±0.095	0.15±0.096	0.13±0.082	0.14±0.093
**CF**	0.16±0.10	0.19±0.13	0.28±0.42	0.18±0.14	0.14±0.11	0.15±0.12
CFK	0.26±0.26	0.25±0.22	0.33±0.35	0.26±0.23	0.19±0.18	0.20±0.17

## References

[1] Durrant-Whyte H., Bailey T. (2006). Simultaneous localization and mapping: Part I. IEEE Robot. Autom. Mag..

[2] Nüchter A., Lingemann K., Hertzberg J., Surmann H. (2007). 6D SLAM – 3D Mapping Outdoor Environments. J. Field Robot. (JFR) Spec. Issue Quant. Perform. Eval. Robot. Intell. Syst..

[3] May S., Dröschel D., Holz D., Fuchs S., Malis E., Nüchter A., Hertzberg J. (2009). 3D Mapping with Time-of-Flight Cameras. J. Field Robot. (JFR) Spec. Issue-Three-Dimens. Mapp..

[4] Li H., Hartley R. The 3D-3D registration problem revisited. Proceedings of the 2007 IEEE 11th International Conference on Computer Vision.

[5] Bellekens B., Spruyt V., Berkvens R., Weyn M. A survey of rigid 3D pointcloud registration algorithms. Proceedings of the AMBIENT 2014: The Fourth International Conference on Ambient Computing, Applications, Services and Technologies.

[6] Marden S., Guivant J. Improving the performance of ICP for real-time applications using an approximate nearest neighbour search. Proceedings of the Australasian Conference on Robotics and Automation.

[7] Besl P.J., McKay N.D. (1992). Method for registration of 3-D shapes. Sensor Fusion IV: Control Paradigms and Data Structures. Int. Soc. Opt. Photonics.

[8] Fantoni S., Castellani U., Fusiello A. Accurate and automatic alignment of range surfaces. Proceedings of the 2012 Second International Conference on 3D Imaging, Modeling, Processing, Visualization & Transmission.

[9] Segal A., Haehnel D., Thrun S. (2009). Generalized-icp. Robot. Sci. Syst..

[10] Rusu R.B., Blodow N., Beetz M. Fast point feature histograms (FPFH) for 3D registration. Proceedings of the 2009 IEEE International Conference on Robotics and Automation.

[11] Tsin Y., Kanade T. (2004). A correlation-based approach to robust point set registration. Proceedings of the European Conference on Computer Visio.

[12] Myronenko A., Song X. (2010). Point set registration: Coherent point drift. IEEE Trans. Pattern Anal. Mach. Intell..

[13] Zheng B., Ishikawa R., Oishi T., Takamatsu J., Ikeuchi K. (2009). A fast registration method using IP and its application to ultrasound image registration. IPSJ Trans. Comput. Vis. Appl..

[14] Rusu R.B., Marton Z.C., Blodow N., Beetz M. Learning informative point classes for the acquisition of object model maps. Proceedings of the 2008 10th International Conference on Control, Automation, Robotics and Vision.

[15] Huber D.F., Hebert M. (2002). Automatic Three-Dimensional Modeling from Reality. Ph.D. Thesis.

[16] Tombari F., Salti S., Di Stefano L. Unique shape context for 3D data description. Proceedings of the ACM workshop on 3D Object Retrieval.

[17] Barnea S., Filin S. (2008). Keypoint based autonomous registration of terrestrial laser point-clouds. ISPRS J. Photogramm. Remote. Sens..

[18] Steder B., Grisetti G., Burgard W. Robust place recognition for 3D range data based on point features. Proceedings of the 2010 IEEE International Conference on Robotics and Automation.

[19] Flint A., Dick A., Van Den Hengel A. Thrift: Local 3d structure recognition. Proceedings of the 9th Biennial Conference of the Australian Pattern Recognition Society on Digital Image Computing Techniques and Applications (DICTA 2007).

[20] Wu C., Clipp B., Li X., Frahm J.M., Pollefeys M. 3D model matching with viewpoint-invariant patches (VIP). Proceedings of the 2008 IEEE Conference on Computer Vision and Pattern Recognition.

[21] Zeng A., Song S., Nießner M., Fisher M., Xiao J., Funkhouser T. 3dmatch: Learning local geometric descriptors from rgb-d reconstructions. Proceedings of the IEEE Conference on Computer Vision and Pattern Recognition.

[22] Gojcic Z., Zhou C., Wegner J.D., Wieser A. The perfect match: 3d point cloud matching with smoothed densities. Proceedings of the IEEE Conference on Computer Vision and Pattern Recognition.

[23] Deng H., Birdal T., Ilic S. Ppfnet: Global context aware local features for robust 3d point matching. Proceedings of the IEEE Conference on Computer Vision and Pattern Recognition, Salt Lake City.

[24] Deng H., Birdal T., Ilic S. Ppf-foldnet: Unsupervised learning of rotation invariant 3d local descriptors. Proceedings of the European Conference on Computer Vision (ECCV).

[25] Bai X., Luo Z., Zhou L., Fu H., Quan L., Tai C.L. D3Feat: Joint Learning of Dense Detection and Description of 3D Local Features. Proceedings of the IEEE/CVF Conference on Computer Vision and Pattern Recognition.

[26] Yew Z.J., Lee G.H. 3DFeat-Net: Weakly supervised local 3D features for point cloud registration. Proceedings of the European Conference on Computer Vision.

[27] Lu F., Chen G., Liu Y., Qu Z., Knoll A. (2020). RSKDD-Net: Random Sample-based Keypoint Detector and Descriptor. arXiv.

[28] Qi C.R., Yi L., Su H., Guibas L.J. Pointnet++: Deep hierarchical feature learning on point sets in a metric space. Proceedings of the Advances in Neural Information Processing Systems.

[29] Maddern W., Pascoe G., Linegar C., Newman P. (2017). 1 year, 1000 km: The Oxford RobotCar dataset. Int. J. Robot. Res..

[30] Geiger A., Lenz P., Urtasun R. Are we ready for autonomous driving? The kitti vision benchmark suite. Proceedings of the 2012 IEEE Conference on Computer Vision and Pattern Recognition.

[31] Yang H., Shi J., Carlone L. (2020). TEASER: Fast and Certifiable Point Cloud Registration. IEEE Trans. Robot..

[32] Huang R., Xu Y., Yao W., Hoegner L., Stilla U. (2020). Robust global registration of point clouds by closed-form solution in the frequency domain. ISPRS J. Photogramm. Remote. Sens..

[33] Qi C.R., Su H., Mo K., Guibas L.J. Pointnet: Deep learning on point sets for 3d classification and segmentation. Proceedings of the IEEE Conference on Computer Vision and Pattern Recognition.

[34] Li J., Lee G.H. Usip: Unsupervised stable interest point detection from 3d point clouds. Proceedings of the IEEE International Conference on Computer Vision.

[35] Fleuret F., Sahbi H. Scale-invariance of support vector machines based on the triangular kernel. Proceedings of the 3rd International Workshop on Statistical and Computational Theories of Vision.

[36] Arun K.S., Huang T.S., Blostein S.D. (1987). Least Square Fitting of Two 3-D Point Sets. IEEE Trans. Pattern Anal. Mach. Intell..

[37] Sorkine O. (2009). Least-squares rigid motion using svd. Tech. Notes.

[38] Larochelle P.M., Murray A.P., Angeles J. (2007). A distance metric for finite sets of rigid-body displacements via the polar decomposition. J. Mech. Des..

[39] Abadi M., Barham P., Chen J., Chen Z., Davis A., Dean J., Devin M., Ghemawat S., Irving G., Isard M. Tensorflow: A system for large-scale machine learning. Proceedings of the 12th {USENIX} Symposium on Operating Systems Design and Implementation ({OSDI} 16).

[40] Järemo Lawin F., Danelljan M., Shahbaz Khan F., Forssén P.E., Felsberg M. Density adaptive point set registration. Proceedings of the IEEE Conference on Computer Vision and Pattern Recognition.

[41] Cignoni P., Callieri M., Corsini M., Dellepiane M., Ganovelli F., Ranzuglia G. Meshlab: An open-source mesh processing tool. Proceedings of the Eurographics ITALIAN Chapter Conference.

[42] Rusu R.B., Cousins S. 3d is here: Point cloud library (pcl). Proceedings of the 2011 IEEE International Conference on Robotics and Automation.

